# Pain in Dental Surgery

**Published:** 1865-02

**Authors:** W. H. Robinson


### Original Essays and Communications.

### PAIN IN DENTAL SURGERY.

BY W. H. ROBINSON.

Read before the Iowa State Dental Association.

IN the discussion of this topic we will first endeavor to show what pain is, and then its relation to Dental Surgery.

Pain is the recognition by the sensorial consciousness of a disagreeable sensation. The sensor nerves being the media through which external impressions are conveyed to the brain. The conscious recognition of these impressions by the mind is sensation, and this sensation will be either painful or pleasant according to the nature of the impression made upon the sensorium; this impression usually being the result produced by external agents coming in contact with the peripheral distribution of the sensor nerves, and by them transmitted to the sensorium and there recognized by the consciousness. The precise means by which external impressions are propagated through the nerve trunk, science has not yet fully revealed in all its complicated minutia. Still we think that it has been unquestionably demonstrated that the agent that transmits with such unerring certainty these impressions, is that all-pervading, invisible, yet potent agent electricity. When an impression is made on some of the extremities, for instance, the fingers, how is that impression conveyed through the nerve media to the brain? Some have supposed that external objects coming in contact with peripheral nerve tissue, caused a vibrating motion that was propaga ed from molecule to molecule throughout the extent of the nerve trunk. But impressions are conveyed that it seems absurd to suppose could produce any vibratory motion in the tissue of the nerve from the nature of such impressions, and the manner in which they are communicated to the nerve. That the nerve fibers are traversed by some agent that transmits impressions, is certain, but to call this agent "nerve-current," or a "vital agent" in the nerve, is not solving the problem. Scientific researches have shown that the electrical condition in no two parts on either side of the human body are the same; while the corresponding parts on opposites are always so, unless when thrown out of balance by some abnormal condition.

The electrical condition of any part seems to depend on the vital operations occurring in it, and that electrical changes occur just as these vital operations are active or dormant. That these molecular states are induced by molecular changes taking place during the vital processes of secretion, assimilation, etc., have been amply proved.

Electrical changes also occur in the muscles during contraction and relaxation, while similar electrical phenomena are found to pervade both sensor and motor nerve trunks. Electrical changes are induced in the nerves by the transmission of sensation, or motive power, through their trunks. This is proved by placing the electrodes of a galvanometer in contact with a nerve trunk. The galvanometer will then indicate the electrical condition between the parts in connection with the electrodes. If these exciting agents be brought in contact with the peripheral fibers of the nerve, electrical changes are indicated in the nerve trunk by the electrical current becoming less and changing its course. This proves that sensation and electrical currents pass through the nerve fibers at the same instant; and that these electrical changes take place when impressions are being transmitted through the nerve trunk. Hence we think that it is a logical inference, that external impressions, or states, are transmitted through the nerve fibers by this *electrical* current. That when an external impression is made on a nerve fiber it induces a certain *electrical* state in the nerve trunk that telegraphs that impression to the sensorium: and, as we have already said, the recognition by the mind of that sensorial impression is sensation. But the impressions received by the mind from the sensorium do not always correspond with the external impressions upon the peripheral distribution of the sensor nerves. This is never absolutely the case, as it is not the external impression that the mind becomes conscious of, but the result of that impression telegraphed through the nerves, *printed* upon the sensorium, and *read* by the mind. Hence the knowledge of external impressions depends upon the following conditions:

*First.* Impressions upon the peripheral distribution of the different nerves. *Second.* Transmission to the sensorium. *Third.* Recognition by the mind. The first and second of the conditions may and often do take place, yet no sensation is experienced until it is recognized by, or conveyed to, the mind. Hence, all that is necessary to produce any *conceivable sensation* is to produce a corresponding impression upon the mind. Consequently, external changes or impressions can give rise to no kind of sensation but what may be the result of internal causes, producing changes or impressions in the trunks of the nerves, or even in the sensorium. In cases of this kind the mind seems to be unable to discern the difference between internal impressions that have no external cause, and those that are produced by tangible external objects. Hence, we have two classes of sensation—those that are produced by external objective causes making impressions upon the sensorium: *these* we call real or objective sensations, and those that are the result of impressions made upon the sensorium without any external cause: these we call imaginary or subjective sensations, because they are the results of some ideational impression produced upon the sensorium and have no reality in the external world. That this class of sensations do exist and produce the same effects as those caused by external objective impressions there can be no doubt. Dr. Carpenter relates an incident that will illustrate this. "A butcher in attempting to hang up a heavy piece of meat, slipped, the hook penetrating his arm so that he himself was suspended. On being examined he was pale, almost pulseless, and expressed himself as suffering acute agony. His arm could not be moved without causing him excessive pain. The pain of removal of the clothes from his arm was so great as to almost deprive him of consciousness. But, alas, when the arm was bared there was not the slightest scratch, nor had it been injured in the least, the hook having penetrated only his coat sleeve." In this case the man not only felt the pain, but experienced all the results that would have followed real lesion of the arm, and to him the pain was real, being the result of a mental impression upon the sensorium without any corresponding physical cause. We see in this and similar cases, that a mental impression that has its origin only in the imagination can be just as forcible in the results as the real or physical impression that has an objective cause. There is but little doubt that the impressions conveyed to the mind through the media of the reason, conscience or imagination, produce the same effects upon the internal consciousness, whether the emotions thus excited in the mind are the result of real or imaginary causes. Similar impressions do not convey the same sensations to different minds. The huntsman is delighted at the bear that terrifies the school boy.

All sensations are greatly affected by the amount of attention we give to them, and by this means can be even produced where before we felt none: for instance, let the mind be specially directed to any particular part of the body as the nose, and we will soon feel sensations of uneasiness there. In cases of this kind it is very difficult to tell whether the impressions that caused this uneasy sensation existed in the nose before we directed our attention to it, or were created by the concentration of the attention upon that particular spot; and *vice versa*, if the attention be wholly withdrawn from any particular organ we are not conscious of moderate impressions upon it causing any sensation. Thus in cases of profound abstraction where the mind is entirely absorbed with other objects, such forcible impressions, as severe wounds and lesions fail to make any impression upon the sensorium, that the mind recognizes, whereas, if the attention be strongly directed to any particular part of the body the slightest impressions made upon it become intensely acute. From this we see, that pain is not necessarily the result of impressions made upon some part of the physical organism; that it is produced by sensorial changes or impressions being communicated to the mind; that these impressions may be objective, produced by the mind becoming conscious of actual impressions on some part of the physical organism, or pain may be subjective, caused by impressions upon the sensorium where these impressions have no external or real cause; but are produced by, and dependent on, certain mental states. Pain is thus experienced whenever the impressions upon the sensorium producing it is communicated to the mind. Consequently, what is commonly called imagination is a reality in the sense that it is the result of a veritable impression upon the sensorium and will be intense or moderate not in proportion to the actual cause, but according to the nature of the sensorial impression.

This fact should ever be borne in mind by the dentist; and, if duly considered, will lead us to exercise reasonable forbearance toward those who receive very forcible sensorial impressions from very minute external causes. The susceptibility of the mind to receive forcible sensorial impressions from minute causes is much augmented by the pathological condition of the general system and by the preconceived and perverted ideas the mind may have formed in regard to such causes. We see this forcibly illustrated in the minor operations of Dental Surgery. Some declaring that the slightest manipulation causes intense agony while others submit to what we call severe operations with little or no suffering. The sensation of pain is the *guardian angel* that our Creator has placed over us to compel us to preserve the integrity of our physical organism; and it is the penalty fixed to a violation of its laws. And although this sensation is peculiarly useful in the normal condition of the body, it may, in regulating many abnormal and diseased conditions, become a serious evil, often being so intense as to cause instant death in surgical operations, that would not otherwise be fatal, and rendering impossible or imperfect many operations that would otherwise be simple and efficacious. Pain in Dental Surgery is a serious annoyance to both patient and practitioner, preventing many from submitting to such Dental operations as are often essential to health and its concomitants, and often causing the Dentist to fail in his best efforts, because the patient will not endure the pain that must necessarily be inflicted in the proper treatment of many of the diseases connected with the dental organs. If it didn't hurt, how easy it would be to extract a tooth, treat alveolar abscess, remove a diseased pulp, or excavate sensitive dentine. How to perform surgical operations without pain has been a problem that many master minds have endeavored to solve, and, to a considerable extent, these efforts have been crowned with success. The goal has not yet been reached, but the prize is not far distant. Some productive intellect will soon seize it, and a crown of gratitude awaits the benefactor who brings forth a perfectly safe and efficient anæsthetic. The anæsthetics we already have are tolerably efficient, and so safe as to justify their use in all capital operations. Yet it is certain that even in the hands of the most prudent administrators they occasionally prove fatal, and if fatality occurs even once in ten thousand cases, it throws a responsibility on the one who gives them in minor operations that demands careful thought before it is assumed. The pain caused by extraction, although severe, is usually of short duration, but the reluctancy and dread with which most persons submit to it has led to numerous expedients for its alleviation. Of course, this, like all other pain, is not experienced in complete general anæsthesia, but in many cases where extraction is advisable, general anæsthesia is not.

Hence, various local expedients have been resorted to, such as congelation of the gums, local anæsthesia by chloroform, and the application of electricity. These local agents have been successful to a limited extent; not so much by inherent virtue in the agent or process as by drawing the attention from the operation to the anæsthetic appliance. And, as we have already shown, pain is not in proportion to the external cause, but is what the sensorial impressions convey to the mind. So it happens, that just so far as any appliance absorbs the attention it mitigates the pain. Alveolar abscess, it is now generally admitted, can only be cured by the removal of the secretive sac that caused it. Where this can be accomplished by therapeutical agents but little pain is produced, but when removed by surgical means, the pain is often considerable, and though seldom as severe as that caused by extraction, yet its being longer in duration, often leads patients to prefer the loss of the tooth to an operation of this kind, and the only advisable anæsthetic we know of in the treatment of alveolar abscess is the communication of a strong sensorial impression to the patient's mind that the tooth is a valuable organ and worth saving even if the operation does cause pain. And lastly, we consider the pain caused by excavating sensitive dentine preparatory to filling; and here we must speak of the formation of dentine. We have two theories on this point, each having adherents among our most scientific odontologists. One is, that the dentine is penetrated by nerve fibers, consequently is vital tissue, but that it possesses such a low grade of vitality that the nerve fibers penetrating, do not, while the dentine is in a healthy state, convey any sensation, but that, in certain diseased and inflammatory states of the dentine, these nerves convey sensation.

The other theory is, that the dentine is not penetrated by nerve fibers, but is composed of tubuli, radiating from the pulp. But the scientific researches of Nasmythe seem to prove that there are no such tubuli, but that what some odontologists mistook for these tubuli are solid fibers, and if so, external compression upon them could not be communicated through them to the pulp.

We hold to the opinion that the dentine is a vital tissue penetrated by nerve fibers, consequently subject to the same changes as the other vital tissues, modified by the peculiar structure and low grade of vitality possessed by it. On this hypothesis the extreme sensibility that is often found to exist in the dentine, is easily accounted for, because exalted sensibility is one of the fundamental indications of inflammation in any tissue. Other indications such as redness and swelling can not take place owing to its structure. None but the serous portion of the blood ordinarily circulates through it, yet in several instances the red corpuscles have been detected in the dentine. All analyses of it prove it to be at least one-fourth animal matter. That the dentine is penetrated by nerve fiber seems to us to be proven beyond the possibility of a doubt from the following observations:

It is sensitive, and sensation is conveyed by nerve fibers. The contact of acids, alkalies and sweets, also changes of temperature cause pain in sensitive dentine. It is easy to see how nerve fibers can receive impressions from these, but difficult to see how these coming in contact with the tubuli of the dentine could produce any impression in the pulp. Excavating might, in some instances, produce impressions on the pulp by compressing the outer ends of the tubuli. But sensation often exists in parts of the dentine that can not possibly have any tubular connection with the pulp. For instance, as in extensive cases of the approximal edges of the incisors, when the decay penetrates the tooth far enough to destroy the tubular connections between the dentine near the cutting edge of the tooth and the pulp; yet in cases of this kind the detached dentine is often more sensitive than that directly in contact with the pulp. But one thing is certain, even if we can not demonstrate the precise means by which the sensation is conveyed in the dentine, the preparation of cavities for filling often causes severe pain, rendering that operation extremely trying to the patience of both patient and dentist, compelling the dentist to perform an imperfect operation or let teeth go unfilled that are very much in need of that operation. Numerous agents are employed for destroying the sensitiveness in dentine. Arsenic, placed in contact with the sensitive surface, is a very efficient agent, but its effects do not stop there, and whenever it is used, great care should be taken not to let it come in contact with any other tissue, as all tissues absorb it, and then death is the result. We think the arsenic should be applied in powder to the sensitive surface, as arsenic in powder is not so readily absorbed as when mixed with some solvent. Chloride of zinc applied in a pledget of cotton will, in from twenty minutes to two hours, destroy sensitiveness in dentine, but its application is so painful that some patients will not endure it; but we think it is less liable to do injury than arsenic, and it has the advantage of being quicker in its effects. A friend of mine had a case in which the dentine was so sensitive that the lady would not endure it to be touched with the excavator, neither would she endure the application of the chloride of zinc. He applied a pledget of cotton saturated with a strong tincture of aconite. This in a short time had the desired effect, so that he was enabled to prepare and fill the cavity without any pain to the patient. If aconite should prove to be usually as efficient as it was in this case it would be a boon of no small value to the dental practitioner. Pain in Dental Surgery deters many of the timorous from submiting to those operations that they know are requisite to health and comfort, and is a serious barrier to perfect operations; its mitigation demands the careful consideration of the philanthropic dentist.

